# The effect of shifts in fish community structure on PCDD/F temporal variability in common guillemot (Baltic Sea)

**DOI:** 10.1007/s13280-026-02387-x

**Published:** 2026-05-03

**Authors:** Yosr Ammar, Jens Olsson, Elena Gorokhova, Martin Sköld, Suzanne Faxneld, Jonas Hentati-Sundberg, Anne L. Soerensen

**Affiliations:** 1 Department of Environmental Monitoring and Research, Swedish Museum of Natural History, P.O. Box 50007, 104 05 Stockholm, Sweden; 2 Department of Environmental Sciences, Faculty of Biological and Environmental Sciences, University of Helsinki, Viikinkaari 9, Helsinki, Finland; 3 Department of Aquatic Resources (SLU Aqua), Swedish University of Agricultural Science, P.O. Box 7018, 750 07 Uppsala, Sweden; 4 Department of Environmental Science, Stockholm University, Svante Arrhenius väg 8, 114 18, Stockholm, Sweden; 5 Department of Mathematics, Stockholm University, Stockholm, Sweden

**Keywords:** Cascading effect, Common guillemot, Environmental changes, Offshore fish community structure, PCDD/Fs

## Abstract

After 1970s, polychlorinated dibenzodioxins and dibenzofurans (PCDD/Fs) concentrations peak in Baltic biota, concentrations started to decline following environmental legislation. However, in common guillemot eggs, this decline plateaued in 1990s, despite continued emissions reductions. Here, we test whether these contrasting trends can be explained by environmental and food web structural changes, including prey availability. Analysing temporal variation in the Central Baltic offshore fish community, including guillemot prey, we identified three structural phases: cod and herring dominance (1976–1986), sprat dominance (1987–2001), and stickleback population increase (2002–2021). We linked them with corresponding phases in PCDD/F trends: a steep decline (− 6.4% yr^−1^), a plateau (0.27% yr^−1^), and a slower decline (− 3.8% yr^−1^). Fish community structure shifts were driven by changes in temperature, salinity, zooplankton size, and fishing pressure. We concluded that climate- and human-driven structural changes in food webs cascaded through trophic levels, affecting PCDD/F concentrations in guillemot eggs.

**Supplementary Information:**

The online version contains supplementary material available at https://doi.org/10.1007/s13280-026-02387-x.

## Introduction

Over the past century, socioeconomic growth has driven major environmental change, leading to three interconnected global crises: climate change, biodiversity loss, and pollution (UNEP 2023). Chemical pollution has become a prominent concern, causing severe environmental and human health issues and altering Earth system processes (Bernhardt et al. 2017; Alpizar et al. 2019). Among pollutants, persistent organic pollutants (POPs) have been recorded in most ocean sediments, water columns, and biota (Alpizar et al. 2019; Willis et al. 2022). POPs bioaccumulate in marine food webs and cause physiological, behavioural, reproductive, and survival challenges in wildlife (e.g. Helander et al. 2002; Vagi et al. 2021; Willis et al. 2022).

Climate change may increase the exposure of biota to POPs and the biomagnification of POPs in food webs with major consequences for marine top predators (Alava et al. 2018; De Wit et al. 2022). The impact of climate change on ecosystems, combined with other anthropogenic pressures, causes complex ecological changes such as alterations in food web structure and species habitats. These processes can directly or indirectly affect POP levels and trends (De Wit et al. 2022). For example, changes in seabirds and marine mammal diet composition can affect their exposure to POPs (De Wit et al. 2022). In East Greenland, changes in polar bear diet resulted in slower declines in adipose concentrations of legacy POPs (McKinney et al. 2013). However, little is known about how these complex ecological processes, shaped by climate change, anthropogenic pressures, food web shifts, and dietary changes, might affect the POPs levels in marine top-predatory birds.

This study focuses on two POP groups: polychlorinated dibenzodioxins (PCDDs) and polychlorinated dibenzofurans (PCDFs), collectively PCDD/Fs. We examine their long-term concentration trends in the marine top-predatory bird, the common guillemot (*Uria aalge*), hereafter referred to as guillemot. PCDD/Fs are groups of hydrophobic and lipophilic organic compounds that bioaccumulate to different degrees in the food web (Isosaari et al. 2004). They are mostly by-products of various industrial, incineration, and combustion processes. PCDD/Fs are regulated under the 1979 Convention on Long-Range Transboundary Air Pollution and included in the original list of 12 POPs under the Stockholm Convention, adopted in 2001.^1^ PCDD/Fs are listed as priority substances under the EU Water Framework Directive (2013/39/EU) and are regulated under the EU Industrial Emissions Directive (2010/75/EU). In the Baltic Sea, major sources include atmospheric deposition, resuspension, and riverine input (McLachlan and Undeman 2020). Their emissions peaked in 1970s when legislation was introduced. Emissions reductions have resulted in declines in concentrations in Baltic Sea biota (Gauss et al. 2020; Soerensen et al. 2024).

**Fig. 1:**
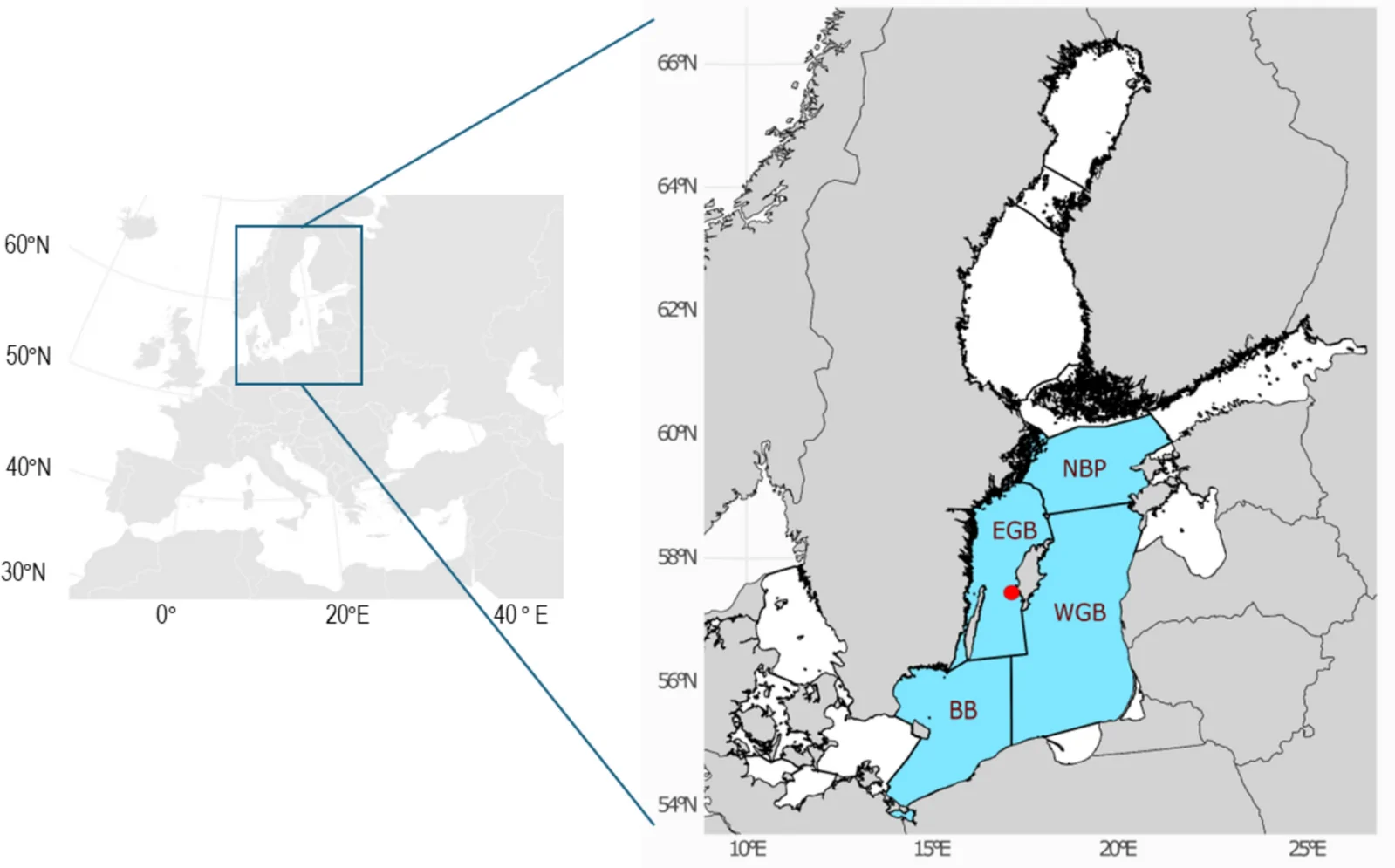
Map of Europe with the Baltic Sea shown in detail. The Central Baltic Sea area in blue includes the HELCOM sub-basins: Northern Baltic Proper (NBP), the Western Gotland Basin (WGB), the Eastern Gotland Basin (EGB), and the Bornholm Basin (BB). The red dot shows the location of Stora Karlsö, the station of the guillemot egg collection

The Baltic Sea guillemot population has undergone substantial changes over the past 150 years. In the late 19th century, it was on the brink of collapse, with only around 20 pairs remaining on Stora Karlsö island (Fig. 1) in 1880 due to extensive hunting and egg collection (Hedgren 1975; Olsson et al. 2000). Since then, the population has increased by an average of 1.8% annually, though the trend has varied, including a decline in 1960s–1970s, probably due to high POPs levels and intense bycatch (Hentati-Sundberg and Olsson 2016). Since 1990s, population growth has been particularly strong, averaging 6.2% annually. Guillemots exhibit high site fidelity, with Stora Karlsö being the biggest Baltic Sea colony (Olsson and Hentati-Sundberg 2017). In the Baltic Sea, guillemots' diet consists primarily of clupeids, especially sprat (*Sprattus sprattus;* until over 90%) and herring, though not exclusively (Lyngs and Durinck 1998; Enekvist 2003; Morkūnė et al. 2016). A recent study, for example, documented that three-spined sticklebacks (*Gasterosteus aculeatus*) account for 27% of adults' diet (Svensson-Israelsson 2025). As high-trophic-level organisms with localized residence, consistent fat content, and low interannual variability in PCDD/F concentrations, guillemots are ideal sentinel species for POPs monitoring in the Baltic Sea (Miller et al. 2014).

Previous studies using Swedish monitoring data reported an unexpected deceleration in the decline rate of PCDD/F concentrations in guillemot eggs and Atlantic herring (*Clupea harengus*) from 1990s to the end of 2010s (Nyberg et al. 2012; Wiberg et al. 2013; Miller et al. 2013, 2014). In herring, this has been linked to slower growth and declining lipid content (Miller et al. 2013). However, the plateau in guillemot eggs remains unexplained. Notably, dioxin air emissions in Sweden have decreased by 73% since 1990, thanks to improved industrial technologies, with the steepest decrease occurring between 1990 and 2000 (Gusev et al. 2023; Naturvårdsverket 2024). This period coincided with the plateau in guillemot eggs PCDD/F concentrations, which supports the hypothesis that additional drivers may influence the observed trends. The limited prey (sprat and herring) PCDD/Fs data available in the Central Baltic Sea (Fig. 1) from the early 2000s onward suggest declining trends (Polak-Juszczak et al. 2022; Soerensen et al. 2024). These data reflect the expected emission-driven decline, offering no insight into the plateau in guillemot eggs.

Besides POPs, the Baltic Sea is affected by climate change, eutrophication, and overfishing, suggesting it as a time machine for understanding climate-induced changes in the global coastal ocean (Reusch et al. 2018). The combined pressures have resulted in multiple regime shifts. These are characterized by abrupt changes in food web structure and function that persist over time and encompass multiple trophic levels (Conversi et al. 2015). In the late 1980s, a shift in dominance from the top predator cod (*Gadus morhua*) to the zooplanktivorous clupeid sprat cascaded through the food web (Alheit et al. 2005; Möllmann et al. 2008, 2009; Tomczak et al. 2022). Since the early 2000s, the mesopredatory fish, the three-spined stickleback, has increased dramatically. This is likely due to favourable environmental conditions combined with declines in its key predators (Olsson et al. 2019; Olin et al. 2022). These food web structural changes involve some of the guillemot’s primary prey species. We investigate whether these changes have contributed to the observed plateau in PCDD/F concentrations in guillemot eggs.

In this study, we hypothesize that, beyond changes in external PCDD/F loads, temporal variability of PCDD/Fs in guillemot eggs is influenced by shifts in environmental conditions (climate and eutrophication) and food web structure. We test this hypothesis by analysing long-term trends in PCDD/F concentrations in guillemot eggs together with fish community structure and environmental data from the Baltic Sea over the past four and a half decades (1975–2021). First, we identify major structural changes in the offshore Baltic Sea fish community, which includes the common guillemot’s main prey species. Then, we relate these changes to temporal trends in PCDD/F concentrations (and toxic equivalents) in guillemot eggs. Finally, we examine environmental and zooplankton community changes as potential drivers of ecosystem variability.

## Materials and methods

### Data description

**PCDD/F congeners** (lipid weight, lw) were measured in guillemot eggs collected from Stora Karlsö in the Central Baltic Sea (Fig. 1) for the Swedish National Monitoring Program for Contaminants in Marine Biota. The data span 1968–2022. Each year, ten eggs were analysed either individually or as homogenates (pooled samples) for seven PCDD and ten PCDF congeners (Table S1, Fig. S1). Analyses were conducted at the Department of Chemistry, Umeå University (see SupInfo). The time series includes a data gap from 1996 to 2001 for all congeners except 2,3,4,7,8-Pentachlorodibenzofuran, the dominant PCDD/F congener (see Fig. S1). Data are available and described in detail in Ammar et al. (2024).

**Toxic Equivalents (TEQs) of PCDD/Fs or TEQ**_**PCDD/Fs**_ (pg/g lw) were calculated using bird-specific Toxicity Equivalency Factors (TEFs) as recommended by Van Den Berg et al. (1998). TEFs account for differences in sensitivity to individual congeners of PCDD/Fs between birds, mammals, and fish. By combining measured congener concentrations with their respective TEFs, the TEQ represents the total toxicity of the mixture. From 1996 to 2001, TEQ_PCDD/Fs_ values were estimated using measured concentrations of 2,3,4,7,8-Pentachlorodibenzofuran. The remaining congeners were inferred from their typical ratios to this compound, and the corresponding TEFs were applied (Haglund et al. 2000).

$$ {\mathrm{WHO}{\mathrm{-}}\textbf{TEQ}}_{{{\textbf{PCDD}}/{\textbf{Fs}}}} = \Sigma \left[ {{\mathrm{PCDD}} \times {\mathrm{TEF}}} \right] + \Sigma \left[ {{\mathrm{PCDF}} \times {\mathrm{TEF}}} \right]\;\left( {{17}\;{\mathrm{congeners}}} \right) $$

**Fish community** data describe the development and characteristics of major Baltic Sea offshore fish species: Atlantic herring (*Clupea harengus*), European sprat (*Sprattus sprattus*), Atlantic cod (*Gadus morhua*), and three-spined stickleback (*Gasterosteus aculeatus*, Table 1). Benthic flatfish was excluded since they do not share dietary overlap with guillemots (Morkūnė et al. 2016). Although guillemots do not prey on cod, they both compete and exert a strong influence on the same primary prey. Data included the following stocks: Central Baltic herring, Baltic sprat, Eastern Baltic cod (ICES 2024), and offshore Baltic stickleback (Bergström et al. 2015). For herring, sprat, and cod, stock variables used were recruitment (R: number of new individuals that survive to enter the adult population in a given time) and spawning stock biomass (SSB: total weight of the mature portion of a fish population that is capable of reproducing). Fishing mortality (F: annual rate of the fish population harvested or killed in fishing) was included as a predictor. These data were taken from the latest ICES Baltic Fisheries Assessment Working Group report (ICES 2024). For the three-spined stickleback, only relative abundance estimates were available (RA: number of individuals per trawl hour) from the Baltic International Acoustic Survey sensu (Bergström et al. 2015). The time series are shown in SupInfo Fig. S2.

**Table 1:** Detailed description of the data (predictor and response variables) used in the analysis time series covering the years 1976 to 2021. All data are averaged over all Central Baltic Sea Basins, except zooplankton, which excludes BB data (Fig. 1). Data sources are: fish stocks from ICES Baltic Sea assessment (ICES 2024), the Baltic International Acoustic Survey sensu (Bergström et al. 2015), zooplankton from ICES DOME database (https://dome.ices.dk/views/Zooplankton.aspx), and environmental data from SharkWeb database (https://sharkweb.smhi.se/)

Group		Data	Full name
Fish community structure		Herring R	Central Baltic herring recruitment (Age 0)
		Sprat R	Baltic sprat recruitment (Age 0)
		Cod R	Eastern Baltic cod recruitment (Age 0)
		Herring SSB	Central Baltic herring spawning stock biomass (tonnes)
		Sprat SSB	Baltic sprat spawning stock biomass (tonnes)
		Cod SSB	Eastern Baltic cod spawning stock biomass (tonnes)
		Stickleback RA	Stickleback relative abundance
Predictors	Fishing mortality	Herring F	Central Baltic herring fishing mortality (tonnes)
		Sprat F	Baltic sprat fishing mortality (tonnes)
		Cod F	Eastern Baltic cod fishing mortality (tonnes)
		Zoo TZB	Summer (May–September) total zooplankton biomass (in mg/m^3^)
		Zoo MS	Summer (May–September) zooplankton mean size (in µg/ind)
	Zooplankton	WSAL	Winter surface (0–10 m) salinity (PSU)
		WBSAL	Winter bottom (> 100 m in EGB, WGB, NBP, and > 90 m in BB) salinity (PSU)
		SuSST	Summer (June–August) sea surface (0–10 m) temperature (°C)
		SpSST	Spring (March–May) sea surface (0–10 m) temperature (°C)
		AuSST	Autumn (September–November) sea surface (0–10 m) temperature (°C)
	Environmental	SuSBT	Summer (June–August) sea bottom (> 100 m in EGB, WGB, NBP, and > 90 m in BB) temperature (°C)
		DIP	Winter (December–February) Dissolved inorganic phosphorus (umol/l)
		DIN	Winter (December–February) Dissolved inorganic nitrogen (umol/l)
		Oxygen	Winter (December–February) deep-water (> 100 m in EGB, WGB, NBP and > 90 m in BB) dissolved oxygen (ml/l)
		Secchi	Summer (June–August) Secchi depth (m)

**Zooplankton** abundance data for May to September (growth season; 1976–2021), including spring and summer blooms, were retrieved from the ICES DOME database^2^ as part of the Baltic Data Flows project.^3^ Zooplankton served as key prey for the fish community during this period. The Zooplankton Mean Size and Total Stock workflow script (Labuce and Gorokhova 2023) was used to calculate two metrics: (i) total zooplankton biomass (Zoo TZB; mg/m^3^) using individual wet weights (Hernroth 1985); for species not included in this list, either measured or calculated individual weights based on length measurements were used. (ii) Mean size of a zooplankter in the community (Zoo MS in µg/ind.) is the average wet mass of an individual within a mesozooplankton community, reflecting its size structure and trophic characteristics. This was derived from abundance and total biomass values for each sampling occasion, from which monthly averages were obtained and subsequently used to calculate summer means (Gorokhova et al. 2016). Zooplankton data from Bornholm Basin (BB, Fig. 1) were excluded due to a substantially shorter time series. The time series are presented in Fig. S3.

**Environmental data** were extracted from the SharkWeb database;^4^ Table 1, Fig. S3). We used the following variables: annual average winter surface (WSAL) and bottom salinity (WBSAL); seasonal average sea surface temperature for summer (SuSST), spring (SpSST), and autumn (AuSST); summer sea bottom temperature (SuSBT); winter average dissolved inorganic nitrogen (DIN) and phosphorus (DIP); winter average deep-water dissolved oxygen (Oxygen); and summer average Secchi depth (Secchi; Fig. S3). These variables are commonly applied in the literature (Tomczak et al. 2022) and capture the main characteristics and changes in the Baltic Sea ecosystem associated with climate change and eutrophication.

### Standardization and integration of time series

PCDD/F congeners with over 10 years of data below the limit of quantification (LOQ) were excluded (Table S1, Fig. S1). This criterion was applied because congeners with predominantly non-quantifiable concentrations provide insufficient quantitative information for reliable trend estimation, and their inclusion could bias the analysis (Helsel 2012).

Fish data were provided as individual time series for each variable. Missing values in the stickleback series were imputed. All fish time series were then normalized. Zooplankton data were normalized, imputed within each basin, and averaged across the Central Baltic Sea (here NBP, WGB, and EGB). Environmental variables, measured at multiple stations, were first averaged within each HELCOM sub-basin (NBP, WGB, EGB, and BB). After imputing missing values within each sub-basin, sub-basin-level time series were combined using equal weighting to derive Central Baltic Sea-wide averages, which were then normalized. Imputations were performed by interpolating between neighbouring observations using the *na.approx* function (Zeileis and Grothendieck 2005) with a maximum interpolation gap of 3 years to balance data continuity with ecological plausibility. All normalizations used mix-max scaling.

All analyses were conducted in R (R Core Team 2022).

### Identification of phases in the fish community structure

To identify shifts in fish community structure, we defined phases characterized by similar fish community compositions (Table 1). We applied a Gaussian mixture model (GMM) using the *mclust* package (Scrucca et al. 2016) to classify phases. The optimal number of clusters was determined objectively using Bayesian Information Criterion (BIC). Unlike distance-based clustering, GMM assigns observations to clusters probabilistically by estimating the likelihood that each observation belongs to each underlying distribution, rather than relying on explicit similarity measures (Fraley and Raftery 2002).

### PCDD/Fs rate of change analysis relative to fish community phases

With the introduction of emission legislation in 1970s, we expect a steady decrease in PCDD/F concentrations in Baltic Sea biota, assuming other environmental conditions remained stable, consistent with trends reported for Baltic Sea sediments (Assefa et al. 2014; Sobek et al. 2015). To detect deviations from the expected pattern, we estimated phase-specific rates of change in PCDD/F and TEQ_PCDD/Fs_ concentrations by fitting linear splines with knots at the phase transitions identified from the GMM-derived fish community shifts. In addition, we estimated the overall rate of change in the TEQ_PCDD/Fs_ time series across all years by fitting linear splines to the log-transformed time series.

### Boosted Regression Tree to identify phase predictors

To identify the drivers associated with each fish community phase (variables explaining differences among phases), we used Boosted Regression Trees (BRT). BRT is a non-parametric regression technique that combines many simple decision trees to capture complex, potentially nonlinear relationships and interactions. Each is sequentially fitted to the residuals of the previous one, without requiring assumptions about the functional form of relationships between predictors (defined as variables with a relative influence on a given phase that exceed what could be expected by chance, e.g. environmental variables) and the response (fish community structure; Elith et al. 2008; Jouffray et al. 2019). Although it does not explicitly model temporal autocorrelation, it can implicitly account for certain types of dependence (Elith et al. 2008).

To run the BRT and quantify the relative influence of predictors within each phase, we followed the procedure described in Jouffray et al. (2019). We used the *gbm.step*() function with default parameters: tree complexity of 5, learning rate of 0.001, and bag fraction of 0.75 (Jouffray et al. 2019). These parameter settings ensure consistency and comparability across models, while balancing complexity and generalization. Alternative parameter combinations were tested but did not yield significant differences. Cross-validation performance was evaluated using deviance, correlation, area under the curve (AUC), and the percentage of explained deviance. All measures indicated robust model performance.

Before the analysis, we applied a multi-step procedure to identify candidate predictors of fish community structure and to prevent excessive collinearity. Predictors with a variance inflation factor (VIF) > 5 were considered collinear and excluded sequentially (Zuur et al. 2010). We also examined pairwise correlations and removed predictors with |*r*|> 0.7, following Jouffray et al. (2019; Fig. S4).

## Results

### Fish community structure phases

**Fig. 2:**
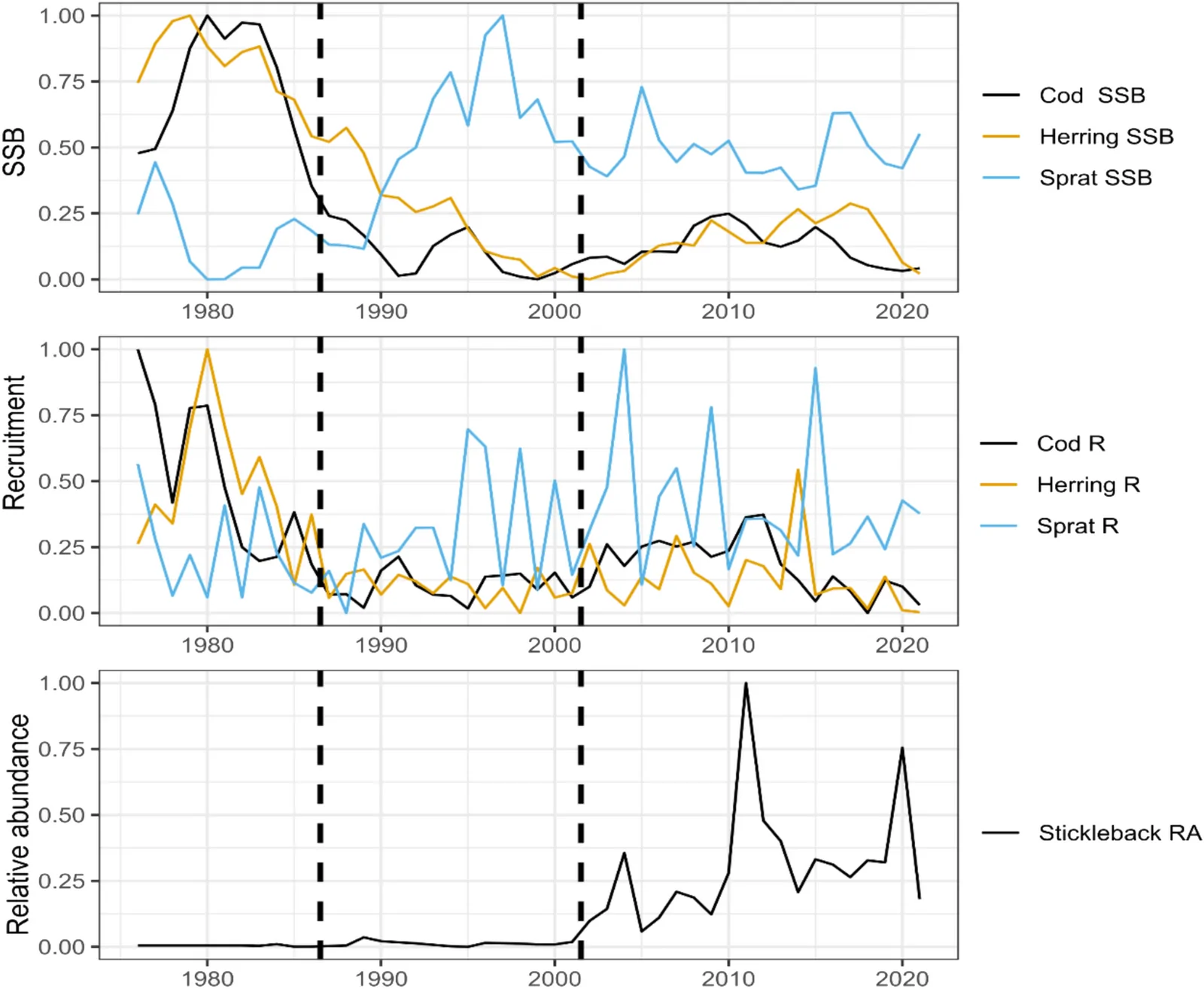
Analysis results of the fish community structure. **a** Fish species time series with sprat, cod, and herring relative stock spawning biomass (SSB) and recruitment (R) in the first and second panels as well as stickleback relative abundance (RA) in the third panel. The dotted black vertical lines indicate the separation between the three phases detected. See Figs. S4 and S5 for further details on the clusters

Figure 2 presents the time series used to assess the fish community structure of the Baltic offshore food web. Cod and herring SSBs and Rs decreased during the late 1980s and early 1990s. In contrast, sprat SSB increased, peaking in the mid-1990s, while R fluctuated over time. Stickleback RA suggested a very low population until the early 2000s, followed by a sharp increase, then a steady upward trend despite large interannual variability.

GMM analysis of changes in fish community structure identified three phases: 1976–1986, 1987–2001, and 2002–2021 (Figs. 2, S4, S5). Cod and herring Rs, cod and sprat SSBs, and stickleback RA primarily drove differences between clusters. Cod SSB was the most influential variable in separating clusters, followed by sprat SSB and cod R. Stickleback RA increased markedly in the most recent phase. Variance estimates suggested the highest variability in cod R and sprat SSB, particularly in the second and third phases.

Overall, the first phase was characterized by high cod and herring SSBs, a relatively low sprat SSB, and very low stickleback RA. The second phase included a sharp decrease in cod and herring SSBs and a rapid increase in sprat SSB. The third phase was marked by a high sprat SSB, low herring and cod SSBs, and a distinct increase in stickleback RA.

### Annual PCDD/F concentrations rate of change during the three phases

**Fig. 3:**
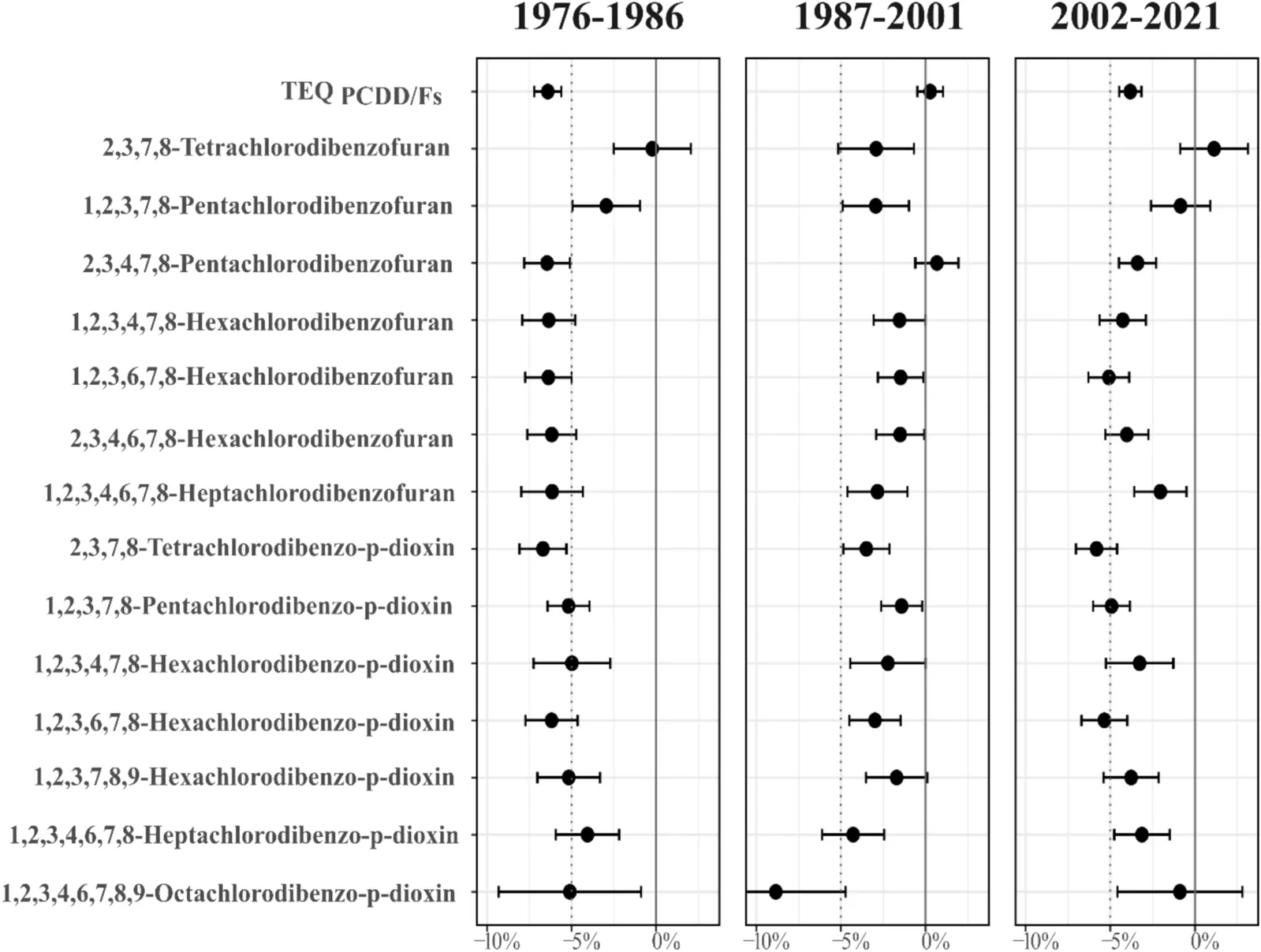
Percentage of average annual change of PCDD/F concentrations in the three identified phases. The error-bar represents the standard deviation of each of the congener’s concentrations

**Fig. 4:**
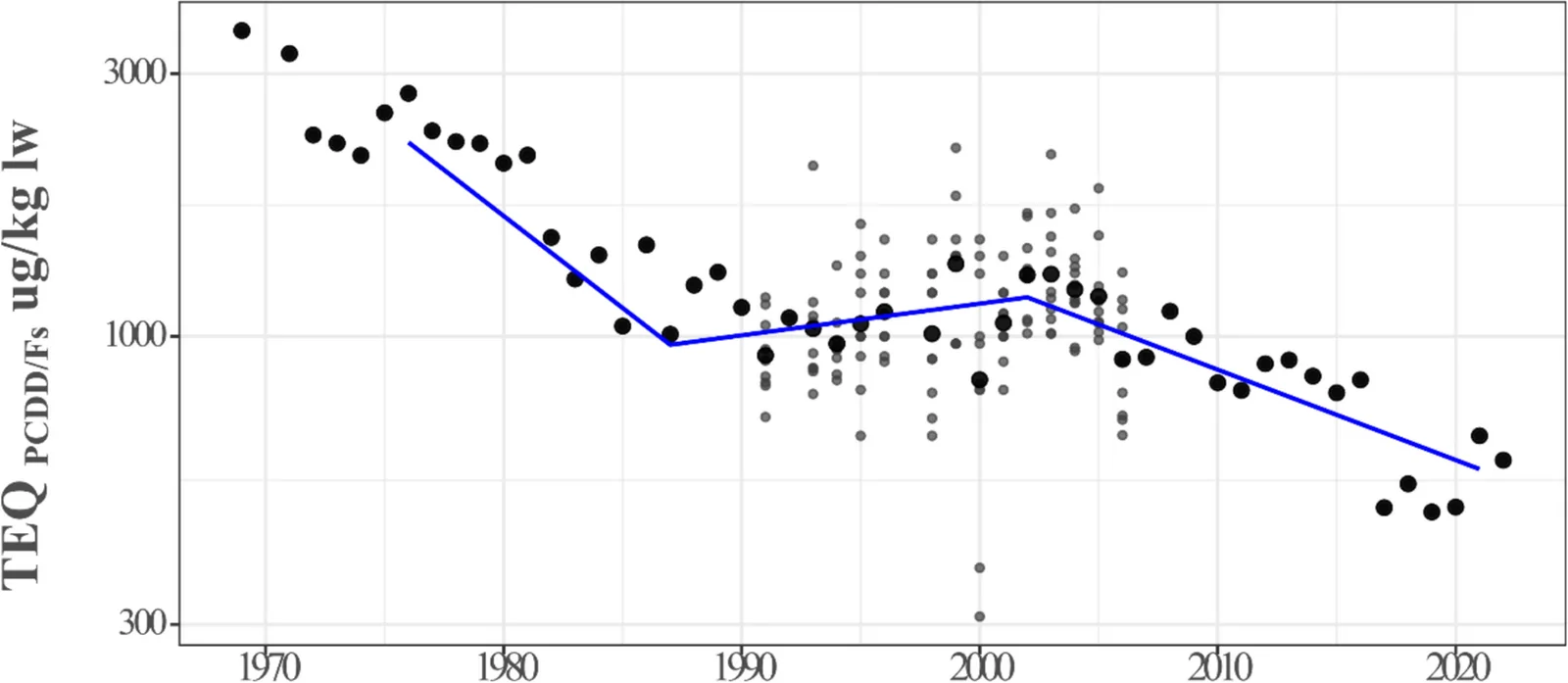
TEQ_PCDD/Fs_ in guillemot eggs from 1968 to 2022. Fitted linear splines to log-transformed series with knots at shifts identified in the fish community structure phase: 1976–1986, 1987–2001, and 2002–2021. Large dots represent annual averages or homogenates, while small dots indicate individual samples

The rate of change in TEQ_PCDD/Fs_ concentrations for the entire time series was − 2.8% yr^−1^. Using the identified fish community structure phases as changepoints (1987 and 2002), we calculated phase-specific rates of change for TEQ_PCDD/Fs_ and individual PCDD/F congeners in guillemot eggs (Fig. 3). During 1976–1986 (cod- and herring-dominated phase), TEQ_PCDD/Fs_ declined at − 6.4% yr^−1^, followed by a near-plateau from 1987 to 2001 (sprat-dominated phase) with a rate of 0.27% yr^−1^. A resumed decline occurred in 2002–2021 (stickleback increase phase) − 3.8% yr^−1^ (Figs. 3, 4).

Most congeners showed the same pattern: a steep decline in the first phase, a slow decrease or plateau (no significant change) in the second, and a moderate decline in the third. Exceptions to these patterns were observed for 2,3,7,8-Tetrachlorodibenzofuran, 1,2,3,7,8-Pentachlorodibenzofuran, and 1,2,3,4,6,7,8,9-Octachlorodibenzo-p-dioxin. As 2,3,4,7,8-Pentachlorodibenzofuran is highly bioaccumulative, occurs at the highest concentrations in the eggs, and has a high TEF (Van Den Berg et al. 1998, Fig. S1), it drove the overall PCDD/F and TEQ rate of change. This congener is mainly a by-product of combustion and incineration, like most PCDFs (Assefa et al. 2019). The individual time series and congener-specific differences are shown in Fig. S1.

### Predictors of change in the Baltic fish community structure

**Fig. 5:**
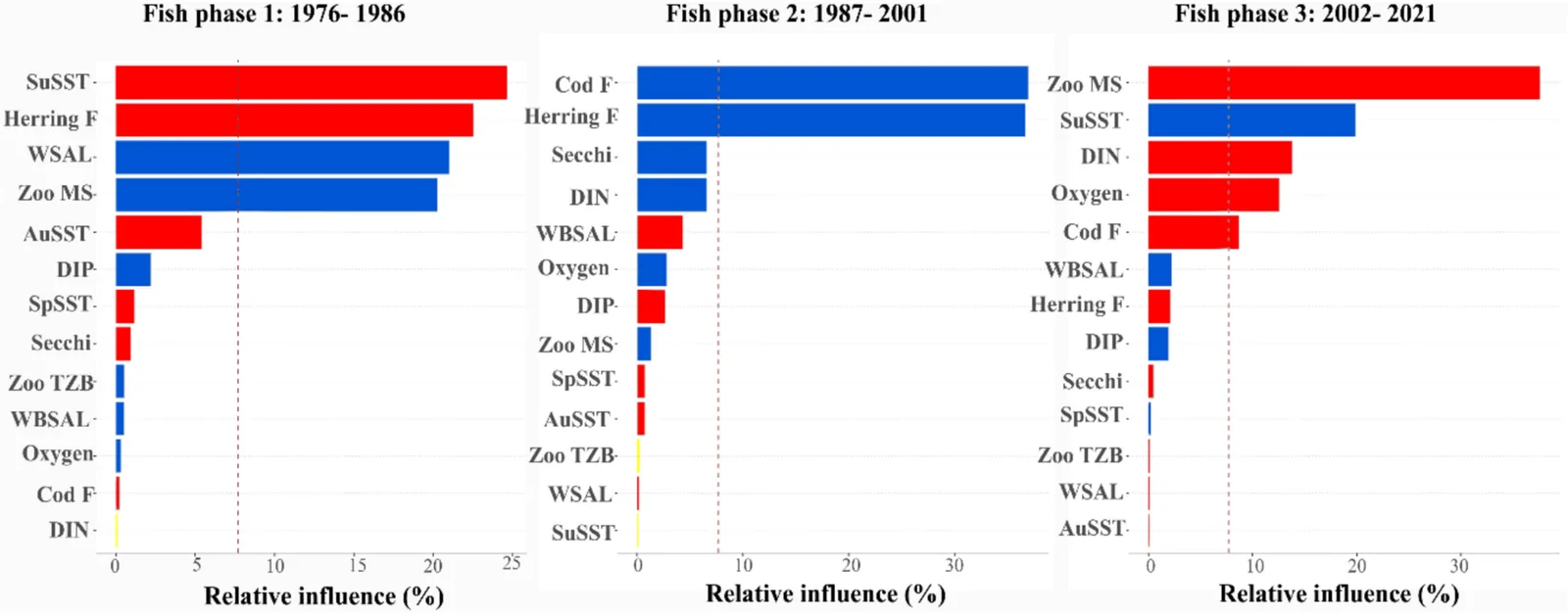
Relative influence of tested predictors associated with each of the three phases identified with BRT analysis. The full name of the variables can be found in Table 1. The colours of the bars reflect the direction of the relationship with blue being positive, red being negative, and yellow indicating a lack of clear direction. The dotted line indicates a relative influence above what could be expected by chance and is considered a potential predictor. See Fig. S7 for information on the direction of the relationship

We used BRT to identify the relative influence of potential predictors driving differences among phases for each of the three fish community structures (Fig. 5). Results indicated robust model performance: explained deviance ranged from 72.43% to 80.16%, correlation coefficients from 0.88 to 0.95, and AUC values from 0.97 to 1. All cross-validation results are provided in Table S2.

During the first phase (1976–1986), BRT identified SuSST, herring F, WSAL, and Zoo MS as potential predictors of the fish community structure. SuSST and herring F exhibited negative relationships with the first phase, suggesting that both variables were low during the cod- and herring-dominated phase when PCDD/Fs declined rapidly. In contrast, WSAL and Zoo MS showed positive relationships with the first phase, indicating that this period was characterized by high salinity and larger-sized zooplankton.

The potential predictors identified for the second phase (1987–2001) were herring and cod Fs, both showing negative relationships with this phase. This suggests that elevated fishing mortality on herring and cod was associated with the fish community structure during this phase, which coincided with declining cod and herring SSBs, increasing sprat SSB, and a concurrent plateau in PCDD/Fs.

During the third phase (2002–2021), elevated SuSST, with a positive relationship, was identified as a potential predictor of the fish community structure. Zoo MS, DIN, oxygen, and cod F also were identified as potential predictors, all exhibiting negative relationships. The third phase was marked by higher stickleback and sprat abundance, low cod, and herring abundances, and resumed PCDD/Fs decline and appeared to be influenced by higher water temperatures, reduced eutrophic conditions, smaller-size zooplankton, and lower cod fishing mortality than the first two phases.

## Discussion

In this study, we analysed environmental and food web structural changes underlying the long-term trends in PCDD/F levels observed in Baltic guillemot eggs over four and a half decades. Earlier observations indicated that while emission controls led to a pronounced initial decline in PCDD/Fs, concentrations plateaued during 1990s, raising concerns about the Baltic Sea’s recovery and long-term environmental implications (Wiberg et al. 2009). We hypothesized that changes in the offshore fish community structure influenced PCDD/F levels in guillemot eggs, resulting in higher-than-expected concentrations. Our findings confirmed that the initial decline of PCDD/F concentrations (− 6.4% yr^−1^ during 1976–1986) plateaued during 1990s (0.27% yr^−1^ during 1987–2001), consistent with previous reports (Nyberg et al. 2012; Wiberg et al. 2013; Miller et al. 2014). However, this plateau was temporary, as the decline resumed at a moderated rate (− 3.8% yr^−1^) after 2002. Below, we discuss potential causes underlying these temporal changes and their implications for future management interventions.

### Structural changes in the Central Baltic Sea offshore fish community

To our knowledge, this is the first study to analyse the offshore Central Baltic Sea fish community structure, including three-spined stickleback, alongside cod, herring, and sprat, and to identify the most recent distinct structural phase (2002–2021). The shift from the first (1976–1986) to the second (1987–2001) phase mirrors the well-documented late 1980s Central Baltic Sea regime shift, during which the system shifted from cod dominance to sprat dominance (Möllmann et al. 2009; Tomczak et al. 2022). Cod, previously exerting top-down control on sprat (Casini et al. 2009), suffered a dramatic biomass decline, leading to a transition in food web dynamics from a benthic- to a pelagic-dominated state (Tomczak et al. 2022). The third phase (2002–2021) is marked by a sharp increase in stickleback relative abundance, alongside high sprat abundance and decline of cod and herring biomass. Stickleback has been suggested to play an increasingly important role in the Baltic offshore pelagic food web (Jakubavičiūtė et al. 2017; Olin et al. 2022) and estimated to contribute to 10% of the Baltic Proper pelagic fish biomass and 40% of the estimated sprat biomass between 2011 and 2014 (Olsson et al. 2019).

### Bottom-up impacts of fish community restructuring on PCDD/Fs in guillemot eggs

When the three identified fish community phases were applied to the guillemot time series, PCDD/F showed phase-dependent differences in concentration declines. We observed a steep decline in the first phase, a plateau in the second, and a slow decline in the third for TEQ_PCDD/Fs_ and most congeners. A few congeners, however, deviated from this pattern, particularly those known to exhibit distinct environmental behaviours (Haglund et al. 2000). While this analysis does not determine the exact timing of shifts in PCDD/F trends, it provides strong evidence of phase-dependent changes in concentration trends, highlighting the fish community dynamics' role in modulating contaminant levels in guillemot eggs.

Our findings indicate that substantial changes in prey availability, resulting from shifts in fish community composition, can cascade through the food web and affect PCDD/F concentrations in the guillemot population. Specifically, changes in sprat biomass, the guillemot´s main prey (Lyngs and Durinck 1998; Enekvist 2003; Morkūnė et al. 2016), may have contributed substantially to the observed plateau in PCDD/F concentrations. The sprat stock, especially the SSB, increased dramatically in 1990s (Fig. 2). Previous research established that sprat population changes cascaded on the common guillemot, affecting chick body mass (Österblom et al. 2006) and fledging success (Kadin et al. 2012). This is the first time this shift has been shown to also affect the trends in contaminant concentrations.

The sprat population increase in 1990s (second phase) intensified the competition within the clupeid community (sprat and herring), leading to density-dependent growth decline for both species (Casini et al. 2006). Sprat weight-at-age dropped by 40% during 1992–1998 and has since remained low, with interannual fluctuations showing no clear trend (ICES 2024). The deterioration in sprat and herring condition also resulted in lower fat content in these prey species available to guillemots compared to earlier years (Røjbek et al. 2014; ICES 2024). In herring, slower growth rates and reduced lipid content have been proposed as the driver of the PCDD/Fs plateau (Miller et al. 2013), suggesting that similar trend was likely occurring in sprat, which also accumulate PCDD/Fs (Vuorinen et al. 2002, 2012). Additionally, guillemot chicks’ feeding rates on clupeids, especially sprat, almost doubled in 1998 and 2002 compared with 1975 (Österblom and Olsson 2002; Enekvist 2003), indicating that guillemots compensated for lower quality prey by increasing feeding rates (Kadin et al. 2016). This compensatory feeding behaviour may have contributed to the PCDD/Fs plateau in eggs through increased consumption of low-quality (low-fat) sprat, which could also be experiencing a deceleration in the decline of PCDD/F concentration trends. A similar pattern has been reported in Baltic salmon, where slow growth, high food competition, and reduced fat content of prey species (i.e. sprat and herring) enhanced the biomagnification of POPs, including PCDD/Fs (Vuorinen et al. 2002, 2012). Hence, our results support the hypothesis proposed by De Wit et al. (2022), that altered diet in seabirds, i.e. changes in prey species and/or prey size, can influence seabirds exposure to POPs (here PCDD/Fs).

During the third phase (2002–2021), sprat SSB remained high, although slightly lower than in 1990s, and sprat condition (weight-at-age) remained poor (ICES 2024). Over the same period, the stickleback population increased, and stickleback may have become an important prey for adult guillemots during the past two decades (Svensson-Israelsson 2025, JHS pers obs.). Although sticklebacks are smaller than sprat, and therefore, less energy-efficient prey, they often occur in high abundance near the surface, making them readily accessible (Carlsen 2022). Stickleback may accumulate less PCDD/Fs than sprat due to their shorter lifespan (3-4 vs. 5-7 years (Ojaveer and Kalejs 2010; Bergström et al. 2015)). Additionally, if sticklebacks have a higher fat content than sprat, guillemots might meet their energy requirements with fewer prey items, potentially reducing PCDD/F uptake. However, data to test this hypothesis are currently lacking. Overall, a dietary shift during the third phase towards increased stickleback consumption could have reduced PCDD/F exposure in guillemots, but this requires further investigation.

### Environmental and anthropogenic pressures shape the Baltic ecosystem

High cod and herring stocks characterized the first fish community phase. Environmental predictors associated with this phase included low SuSST, high WSAL, large-sized zooplankton, and low herring F. Cooler temperatures and higher salinity favoured larger copepods, such as *Pseudocalanus acuspes*, which are important prey for cod larvae and herring (Möllmann et al. 2008). However, across the study period, sea surface temperatures increased steadily, while salinity declined after the second phase, both consistent with long-term climate change trends (HELCOM 2023). These changes favoured sprat and smaller copepods (e.g. *Acartia* spp.) and disadvantaged cod, the main predator of sprat (Alheit et al. 2005; Möllmann et al. 2008; Ojaveer and Kalejs 2010). Since 1990s, Zoo MS has declined due to predation pressure, shifts in phytoplankton composition, and decreasing salinity, reducing fish prey availability and quality for fish (Gorokhova et al. 2016; HELCOM 2023). This decline reflects an increased contribution of small cladocerans (e.g. *Bosmina*) and rotifers (e.g. *Keratella*) at the expense of copepods, which lowers the mass-specific lipid content of zooplankton (Gorokhova 2019). Reduced prey quality likely constrained herring and sprat growth, as their competition for food and space intensified (Diekmann et al. 2010).

In the second sprat-dominated phase, cod and herring Fs were the potential predictors of sprat increase, and cod and herring decrease. Intense herring fishing pressure has been previously identified as a driver of herring stock decline, alongside reduced growth and lower recruitment success (Diekmann et al. 2010). The cod fishery has been one of the main anthropogenic activities affecting cod biomass, with a cascading effect on the entire central Baltic food web (Casini et al. 2008; Möllmann et al. 2009; Tomczak et al. 2022). The declines in cod reduced predation pressure and allowed the sprat stock to flourish.

The third phase is marked by substantial stickleback increase, alongside a relatively stable sprat stock. This phase is associated with predictors including smaller-sized zooplankton, lower DIN and oxygen concentrations, reduced cod F, and rising SuSST. Stickleback thrived during this phase, benefiting from the rise of small, low-lipid zooplankton, particularly cladocerans (Lankov et al. 2010). Sticklebacks consume more cladocerans than herring and sprat, thereby reducing food competition and further enhancing their competitive advantage (Lankov et al. 2010; Jakubavičiūtė et al. 2017; Olin et al. 2022). Warmer water temperatures have likely benefited their ingestion and growth rates, as they migrate from the coast to the open sea for feeding at the end of summer (Lefébure et al. 2014; Bergström et al. 2015). Although tolerant of eutrophic conditions, stickleback growth improves in less eutrophic waters (Olin et al. 2022), and the recent DIN decline may have further facilitated their expansion. Finally, cod stock collapse, driven by fishing pressure alongside decreasing salinity and oxygen levels from reduced North Sea inflows, further contributed to fish community structural changes (Tomczak et al. 2022; Hansson and Viktorsson 2023) (Fig. S8) and led to the cod fishery closure (ICES 2024). Consequently, declines in cod and herring stocks, both stickleback predators, combined with temperature and salinity changes, led to favourable conditions for stickleback to thrive (Olin et al. 2022; Donadi et al. 2024). This may have contributed to ongoing food web restructuring, further altering dietary exposure to contaminants.

## Conclusion and management implications

Food webs structural changes driven by climate change and anthropogenic pressures, including management, can cascade through trophic levels, ultimately affecting bioaccumulation patterns in top predators, such as fish-eating birds. Our results suggest that the plateau in PCDD/F concentrations in guillemots, despite declining emissions, resulted from prey composition, growth, and condition changes mediated by food web restructuring driven by environmental change and fisheries management. This highlights the importance of fisheries management approaches that consider both direct and indirect impacts, given their cascading effects in ecosystems. When, for example, setting single-species quotas, it is necessary to consider broader ecological consequences, including unintended impacts on contaminant transfer within the food web. As this study demonstrated, the abundance, growth, and condition of targeted fish stocks can counteract management efforts to reduce contaminant loads in ecosystems, stressing the need for holistic ecosystem-based approaches to fisheries and environmental management.

Unlike for humans and fish, environmental quality standards for PCDD/Fs in birds are poorly defined, and specific thresholds for adverse effects remain uncertain. Hence, we cannot assess the toxicological significance of TEQ_PCDD/Fs_ values reported for guillemot eggs in this study (Fig. 4). Nonetheless, POPs presence can weaken food web resilience, even at concentrations below established toxicity thresholds and in the absence of observable population declines (Wolff et al. 2019). It is therefore imperative to continue efforts to reduce PCDD/F contamination and to adopt ecosystem-based approaches to prevent the reversal of these reductions and safeguard ecosystem resilience amid ongoing environmental changes.

Over the past 20 years, PCDD/Fs emissions remained low in Europe but rose in Africa and Asia (Lei et al. 2021). Currently, PCDD/Fs occur in 60% of Arctic and Antarctic marine sediments, and POP concentrations in polar regions are comparable to those in the southern Baltic Sea (Kobusińska et al. 2020). Climate change can exacerbate biota exposure to POPs via secondary reemission, sea ice remobilization, and increased run-off (Borgå et al. 2022). Furthermore, climate- and human-driven food web changes can prolong biota exposure, as demonstrated in this study. These trends raise questions about the persistence of PCDD/Fs and other POPs globally, their impacts on marine ecosystems and top predators, and the effectiveness of current mitigation efforts. Nonetheless, chemical pollution remains largely overlooked in global change analyses (Bernhardt et al. 2017). There is an urgent need for interdisciplinary approaches that integrate pollution with the other major global crises (climate change and biodiversity loss) to evaluate their cumulative impacts across ecosystems and develop effective mitigation strategies.

## Supplementary Information

Below is the link to the electronic supplementary material.

Supplementary file1 (PDF 1761 KB)

## Data Availability

All data used in this manuscript are open-access and can be accessed through the references provided in the manuscript.

## Footnotes

1 http://www.pops.int.

2 https://dome.ices.dk/views/Zooplankton.aspx.

3 https://balticdataflows.helcom.fi.

4 https://sharkweb.smhi.se/.
