# Supplementary material for: The effect of shifts in fish community structure on PCDD/F temporal variability in common guillemot (Baltic Sea)
Source: Ambio. 2026 May 3;55(11):2750–64. doi: 10.1007/s13280-026-02387-x (PMC13661839; doi:10.1007/s13280-026-02387-x)
Supplement: Supplementary file 1 [file 13280_2026_2387_MOESM1_ESM.pdf]

Supplementary information

The effect of Shifts in Fish Community Structure on PCDD/F temporal variability in common guillemot (Baltic Sea).

1. Data: Time series description

*Table.S1: PCDD/Fs analyzed in guillemot eggs from Stora Karlsö station in the Baltic Sea. Data sources are: PCDD/Fs from the Swedish National Monitoring Program for Marine Biota (Ammar et al. 2024). * Indicates congeners excluded from the analyses.*

**PCDD/Fs** (pg/g lw)	**Full name**
TCDF	2,3,7,8-Tetrachlorodibenzofuran
TCDD	2,3,7,8-Tetrachlorodibenzo-p-dioxin
PECDF1	1,2,3,7,8-Pentachlorodibenzofuran
PECDF2	2,3,4,7,8-Pentachlorodibenzofuran
PECDD	1,2,3,7,8-Pentachlorodibenzo-p-dioxin
HXCDF1	1,2,3,4,7,8-Hexachlorodibenzofuran
HXCDF2	1,2,3,6,7,8-Hexachlorodibenzofuran
HXCDF3	2,3,4,6,7,8-Hexachlorodibenzofuran
HXCDF4*	1,2,3,7,8,9-Hexachlorodibenzofuran
HXCDD1	1,2,3,4,7,8-Hexachlorodibenzo-p-dioxin
HXCDD2	1,2,3,6,7,8-Hexachlorodibenzo-p-dioxin
HXCDD3	1,2,3,7,8,9-Hexachlorodibenzo-p-dioxin
HPCDF1	1,2,3,4,6,7,8-Heptachlorodibenzofuran
HPCDF2*	1,2,3,4,7,8,9-Heptachlorodibenzofuran
HPCDD	1,2,3,4,6,7,8-Heptachlorodibenzo-p-dioxin
OCDF*	1,2,3,4,6,7,8,9-Octachlorodibenzofuran
OCDD	1,2,3,4,6,7,8,9-Octachlorodibenzo-p-dioxin

Table.S1 shows the 17 congeners analyzed within the Swedish Monitoring Program for Contaminants in Marine Biota (Ammar et al. 2024). PCDD/Fs analyses were carried out at the Department of Chemistry, Umeå University. The extraction method was described by Wiberg et al. (1998), the clean-up method by Danielsson et al. (2005), and the instrumental analysis using Gas Chromatography (GC) coupled to a high-resolution mass spectrometer (GC-HRMS) following Liljelind et al. (2003). The laboratory was participating in the annual FOOD intercalibration rounds, and was including laboratory CRM (salmon tissue) with each set of samples.

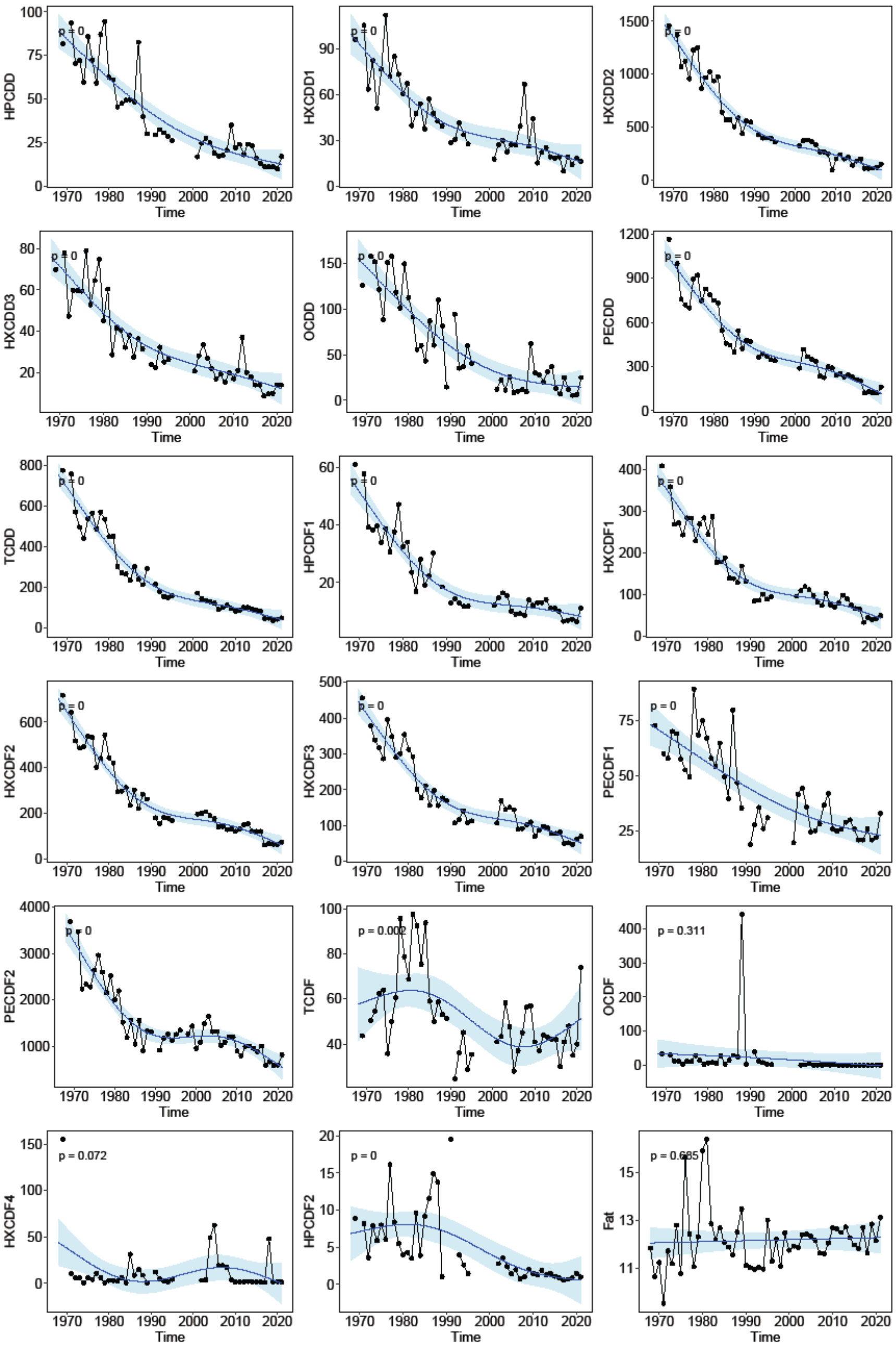

Figure.S1: Time series of PCDD/F concentrations in guillemot eggs between 1968 and 2021 as well as the fat % in the eggs with trend lines fitted using smoothing splines in a Generalized Additive Model (GAM). TCDF: 2,3,7,8-Tetrachlorodibenzofuran, TCDD: 2,3,7,8-Tetrachlorodibenzo-p-dioxin, PECDF1: 1,2,3,7,8-Pentachlorodibenzofuran, PECDF2: 2,3,4,7,8-Pentachlorodibenzofuran, PECDD: 1,2,3,7,8-Pentachlorodibenzo-p-dioxin, HXCDF1: 1,2,3,4,7,8-Hexachlorodibenzofuran, HXCDF2: 1,2,3,6,7,8-Hexachlorodibenzofuran, HXCDF3: 2,3,4,6,7,8-Hexachlorodibenzofuran, HXCDF4: 1,2,3,7,8,9-Hexachlorodibenzofuran, HXCDD1: 1,2,3,4,7,8-Hexachlorodibenzo-p-dioxin, HXCDD2: 1,2,3,6,7,8-Hexachlorodibenzo-p-dioxin, HXCDD3: 1,2,3,7,8,9-Hexachlorodibenzo-p-dioxin, HPCDF1: 1,2,3,4,6,7,8-Heptachlorodibenzofuran, HPCDF2*: 1,2,3,4,7,8,9-Heptachlorodibenzofuran, HPCDD: 1,2,3,4,6,7,8-Heptachlorodibenzo-p-dioxin, OCDF*: 1,2,3,4,6,7,8,9-Octachlorodibenzofuran, OCDD: 1,2,3,4,6,7,8,9-Octachlorodibenzo-p-dioxin. * Indicates congeners excluded from the analyses. Data sources are: PCDD/Fs from the Swedish National Monitoring Program For Marine Biota (Ammar et al. 2024).*

Most congener time series in Figure.S1 showed an overall downward trend with a plateau in the 1990s and 2000s. OCDD and TCDF are known to behave differently from other PCDD/F congeners (Haglund

et al., 2000). HXCDF4, OCDF, and HPCDF2 time series were excluded from the analyses as values before 1995 are in majority below the limit of quantification (LOQ). The fat percentage (Fat %) fluctuated greatly before the mid-1980s, but became more stable with lower variability afterward.

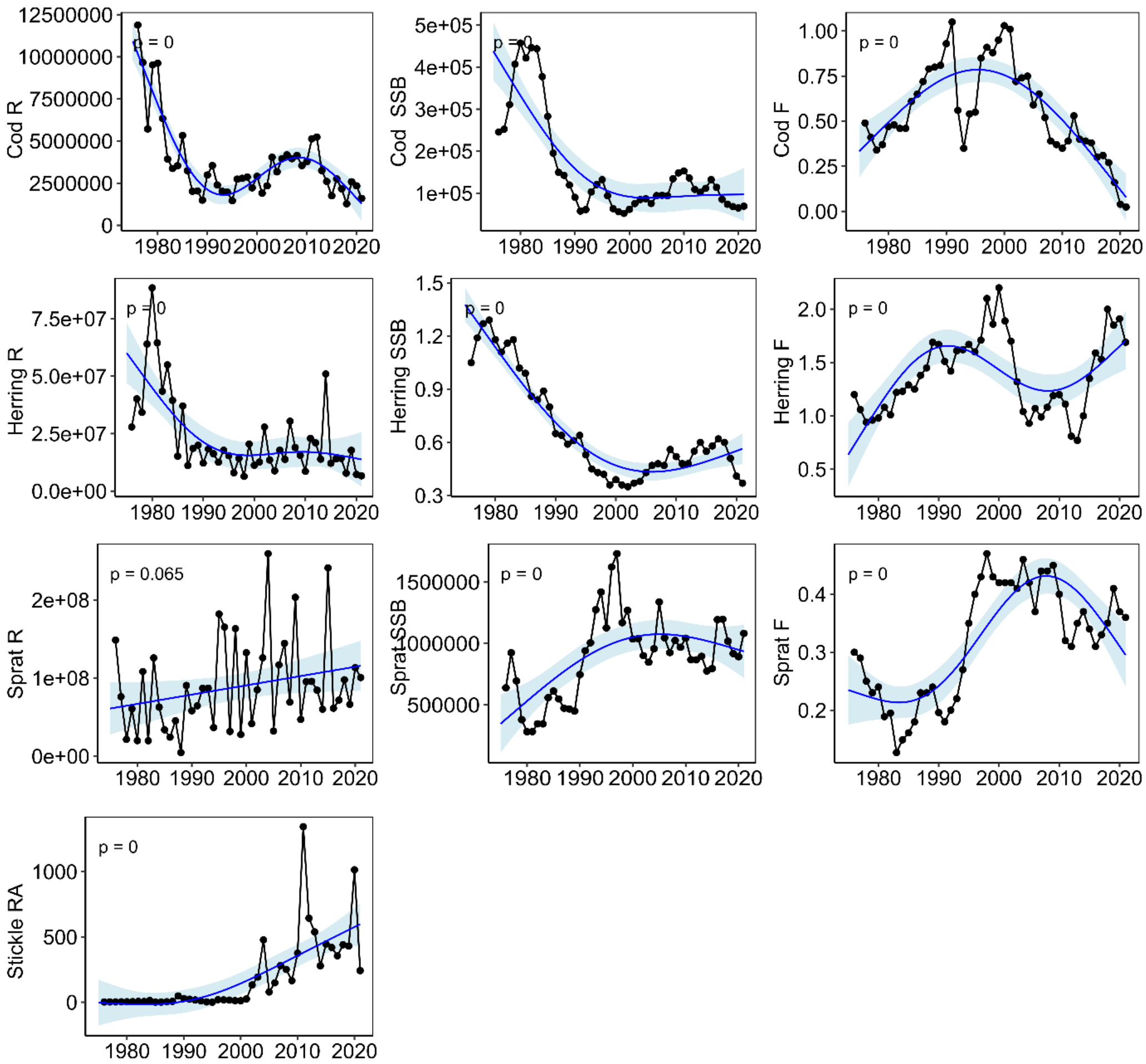

Figure.S2: Fisheries time series with trend lines fitted using smoothing splines in a Generalized Additive Model (GAM) for cod, herring and sprat recruitment (R), spawning stock biomass (SSB) and fishing mortality (F) as well as stickleback relative abundance (Stickle RA).

Cod recruitment decreased to a low value in the early 1990s, rose slightly in the late 2000s, and then decreased again until the end of the time series (Figure.S2). Cod SSB peaked in the early 1980s dropped in the late 1980s and remained low thereafter. Cod fishing mortality increased gradually until the mid-1990s, dropped for a few years and in the 1980s, rose again to a high value in the early 2000s, then decreased gradually thereafter.

Herring recruitment peaked in the early 1980s, decreased in the mid-1980s, and remained at similar levels until the end of the time series (Figure.S2). Herring spawning stock biomass decreased gradually until the early 2000s when it slightly increased, then it dropped again in the late 2010s. Herring fishing mortality increased gradually until the early 2000s, declined between the early 2000s and early 2010s, then increased again in the late 2010s.

Sprat recruitment was highly variable with an overall upward trend (Figure.S2). Sprat spawning biomass increased gradually to reach the highest values in the 1990s, then decreased slightly in the 2000s, and

maintained a stable level afterwards. Sprat fishing mortality was low at the beginning of the time series, increased in the mid-1990s and remained high afterwards.
Stickleback relative abundance was null at the beginning of the time series and started increasing in the mid-2000s with high fluctuations (Figure.S2).

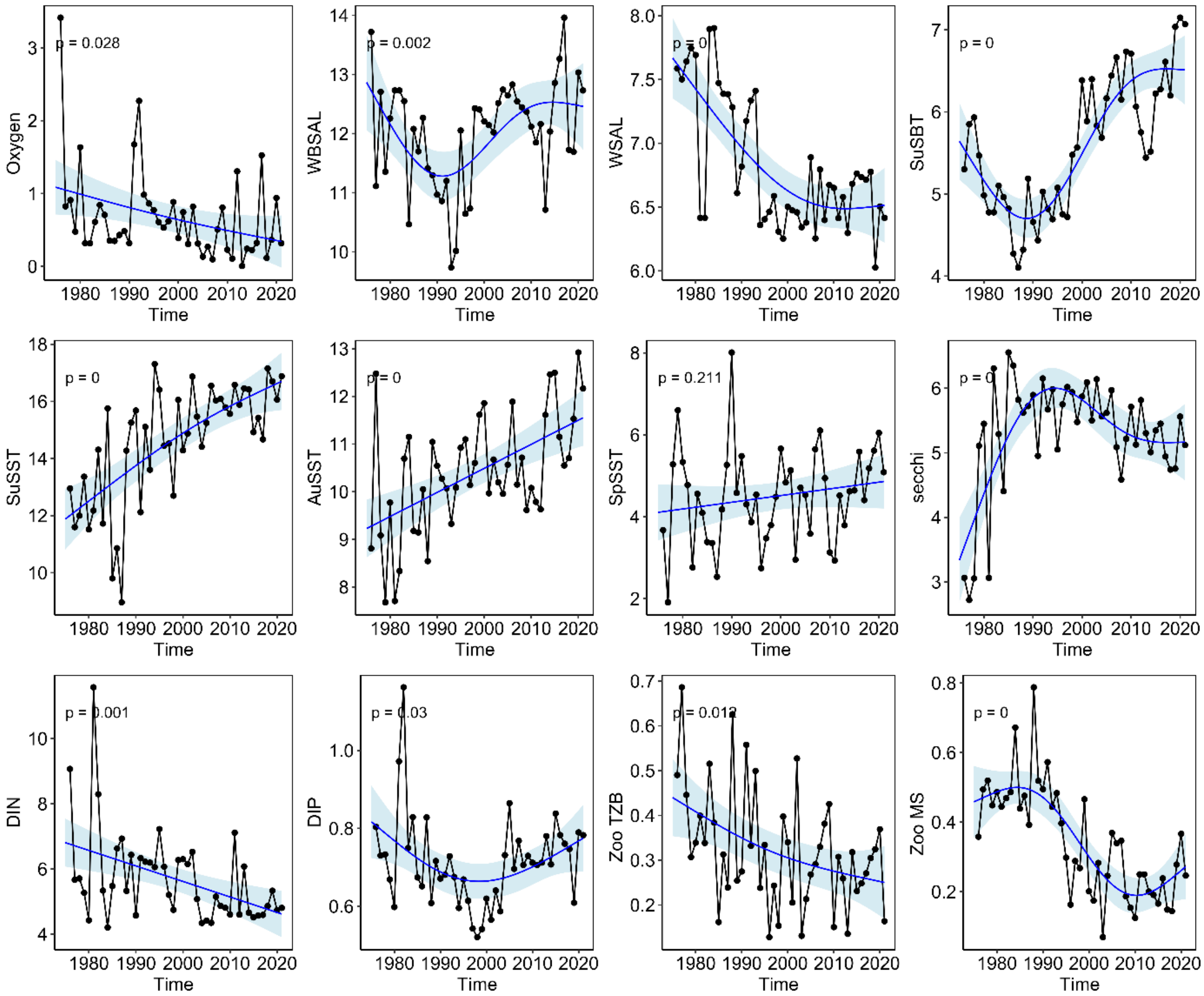

Figure.S3: Time series of environmental data with trend lines fitted using smoothing splines in a Generalized Additive Model (GAM): oxygen, winter bottom salinity (WBSAL), winter salinity (WSAL), summer bottom temperature (SuSBT), summer sea surface temperature (SuSST), autumn sea surface temperature (AuSST), spring sea surface temperature (SpSST), Secchi depth, dissolved organic nitrogen (DIN), and dissolved organic phosphorus (DIP), and of zooplankton data: total zooplankton biomass (Zoo TZB) and zooplankton mean size (Zoo MS).

Oxygen showed an overall downward trend with some fluctuations and close to hypoxic conditions towards the end of the time series (Figure.S4). Winter bottom salinity declined from 13.5 to less than 10 psu, reached its lowest in the early 1990s, and increased in the early 2000s to around 12.5 psu. Winter salinity fluctuates with a downward trend from around 7.5 to 6.5 psu. Summer bottom temperature decreased at the beginning of the time series with the lowest values found in the late 1980s, then increased gradually until the end of the time series. Summer, autumn and spring sea surface temperature had an overall upward trend with high fluctuations with respective 6 °C, 3 °C and 1 °C increase between the start and end of the time series. Secchi depth varies between 3 and 6 meters until the early 1990s then slowly decreases and fluctuates between 5 and 6 meters throughout the rest of the time series. Dissolved inorganic nitrogen had an overall downward trend. Dissolved inorganic phosphorus showed a downward trend until the early 2000s then started increasing slightly.

Total zooplankton biomass showed an overall downward trend with high fluctuations. Zooplankton mean size has decrease starting from the early 1990s.

2. Phase identification

GMM analysis identified three distinct phases in the fish community structure using a **VVI model** (diagonal covariance with varying volume and shape, Figure.S4), as selected based on Bayesian Information Criterion (BIC). The optimal model included **three clusters** with respective sample sizes of **11, 15, and 20 observations** (Figure.S5). The log-likelihood of the final model was **-3949.418**, with a **BIC of -8090.268** and an Integrated Complete Likelihood (ICL) of **-8090.337**, indicating a stable classification. The centroids of the clusters revealed clear temporal shifts, with the mean years for each phase centered around **1981, 1994, and 2011**, respectively. Classification uncertainty was minimal, as reflected in the cluster membership probabilities, where each observation was strongly assigned to one cluster (posterior probabilities close to 1).

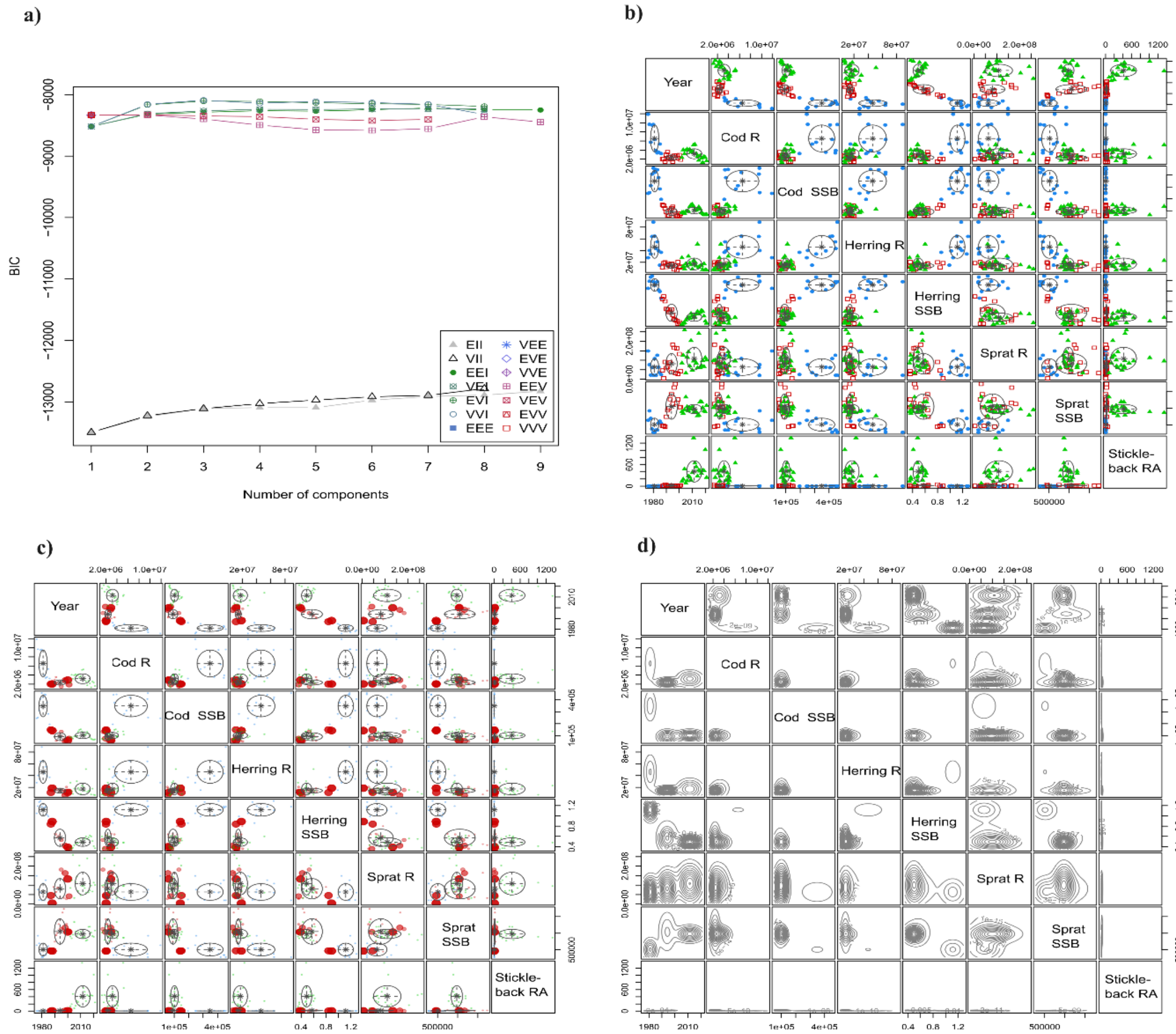

Figure.S4: Model-based clustering analysis: a) Bayesian Information Criterion (BIC) showing model fit for different numbers of clusters, used for model selection with the chosen VVI model; b) Classification of data points into clusters based on the selected model; c) Uncertainty of classification, displaying the posterior probability of each data point belonging to a particular cluster; d) Density of the data points within each cluster, illustrating the distribution of data across different groups.

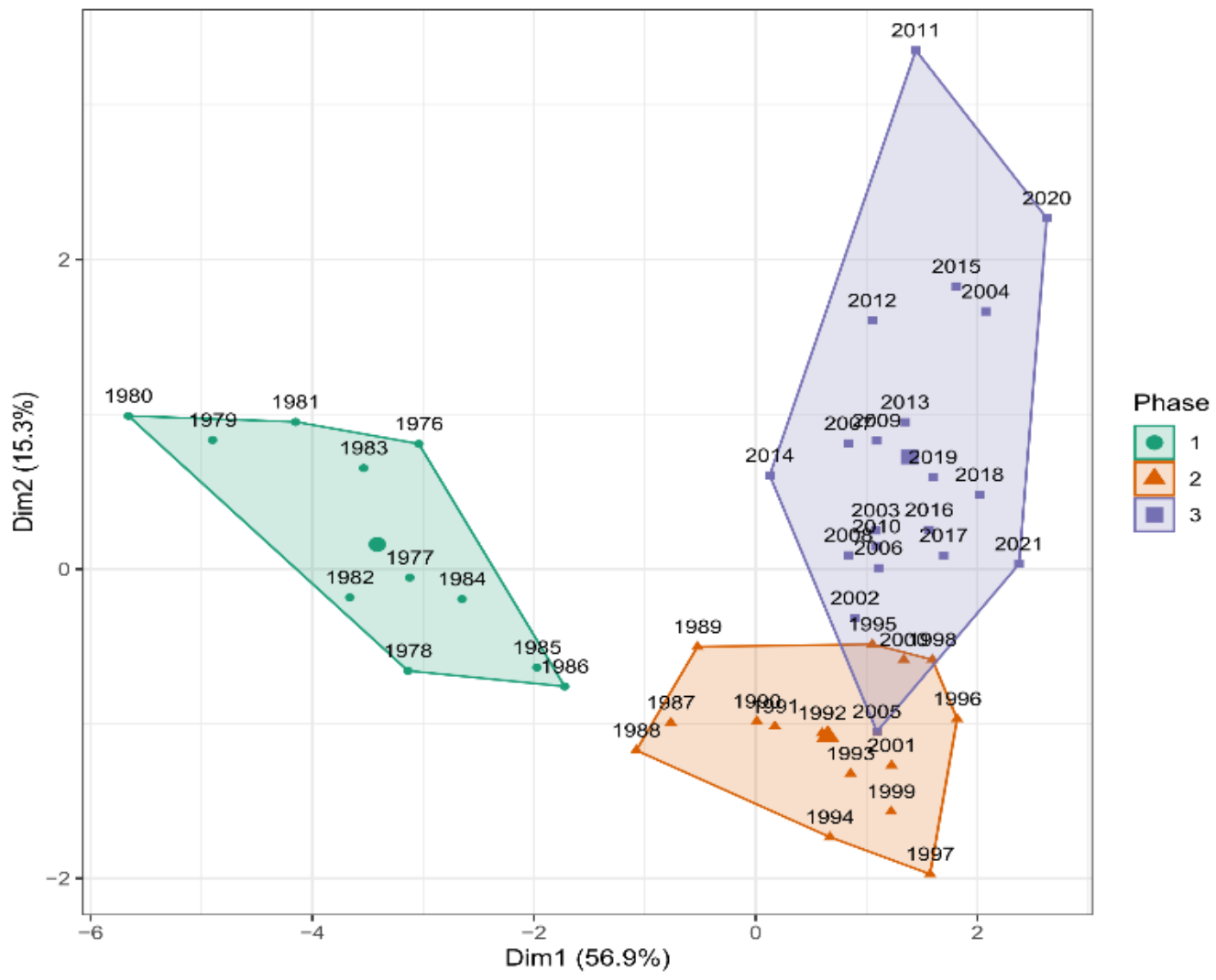

Figure.S5: Cluster plot of fish community structure data based on the classification from GMM model-based clustering. Data points are coloured according to their assigned clusters, with convex hulls outlining each cluster.

3. Data selection

To analyze the fish community structure drivers, we tested all environmental and zooplankton data in addition to fishing mortality of sprat, cod and herring. After variable selection using VIF/correlation check, we eliminated summer bottom temperature, which was highly correlated to winter bottom salinity. The resulting correlations between predictors are shown in Figure.S6.

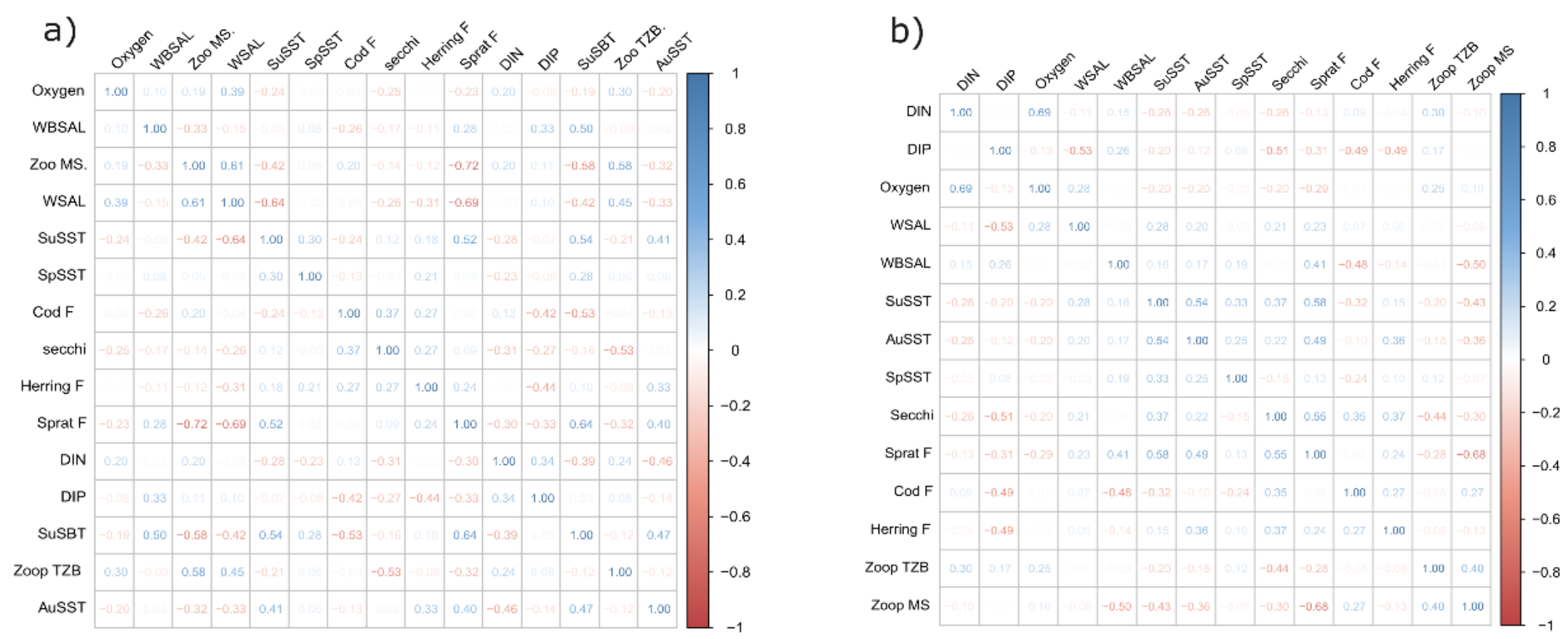

Figure.S6: Correlation plot of predictors used for the fish community structure BRT analysis a) before and b) after the selection process. Prior to correlation analysis, Variance Inflation Factor (VIF) were applied for variable selections. The correlations chosen are <0.7.

4. Boosted regression tree results

3.1. Performance metrics

The BRT analysis was conducted for three distinct fish community structure phases, using the model settings: tree complexity = 5, learning rate = 0.001, and bag fraction = 0.75. The results include several performance metrics for both the training and cross-validation sets. The performance metrics include the Total Deviance (the total variation in the data), Residual Deviance (unexplained variation in the data

after fitting the model), Correlation (strength of the relationship between the predicted and observed values), Area Under the Curve (AUC), and Percentage Explained Deviance. The cross-validation (CV) statistics include CV Deviance, CV Correlation, CV AUC, and CV Percentage of Explained Deviance.

The BRT model performs well on training and cross-validation sets, with high correlation, AUC, and percentage of explained deviance. The model explains a large portion of the variance in the fish community structure in Table.S2 for all phases.

Table.S2: Performance metrics and cross-validation (CV) statistics for the fish community structure boosted regression tree (BRT) analysis. Tree complexity = 5, learning rate = 0.001, bag fraction = 0.75.

Performance metrics and cross validation	Phase 1	Phase 2	Phase 3
Total Deviance	1.10	1.26	1.37
Residual Deviance	0.02	0.04	0.02
Correlation	1	1	1
AUC	1	1	1
Percentage of Explained Deviance	98.14	96.92	98.72
CV Deviance	0.3	0.31	0.27
CV Correlation	0.88	0.95	0.93
CV AUC	0.97	1	1
CV Percentage of Explained Deviance	72.43	75.39	80.16

3.2. Partial dependency plots

Figure.S7 shows the partial dependency plots that provide insights into the chosen predictors contribute to shifts in the system. These plots were used to identify the direction of the relationship for each predictor in different phases.

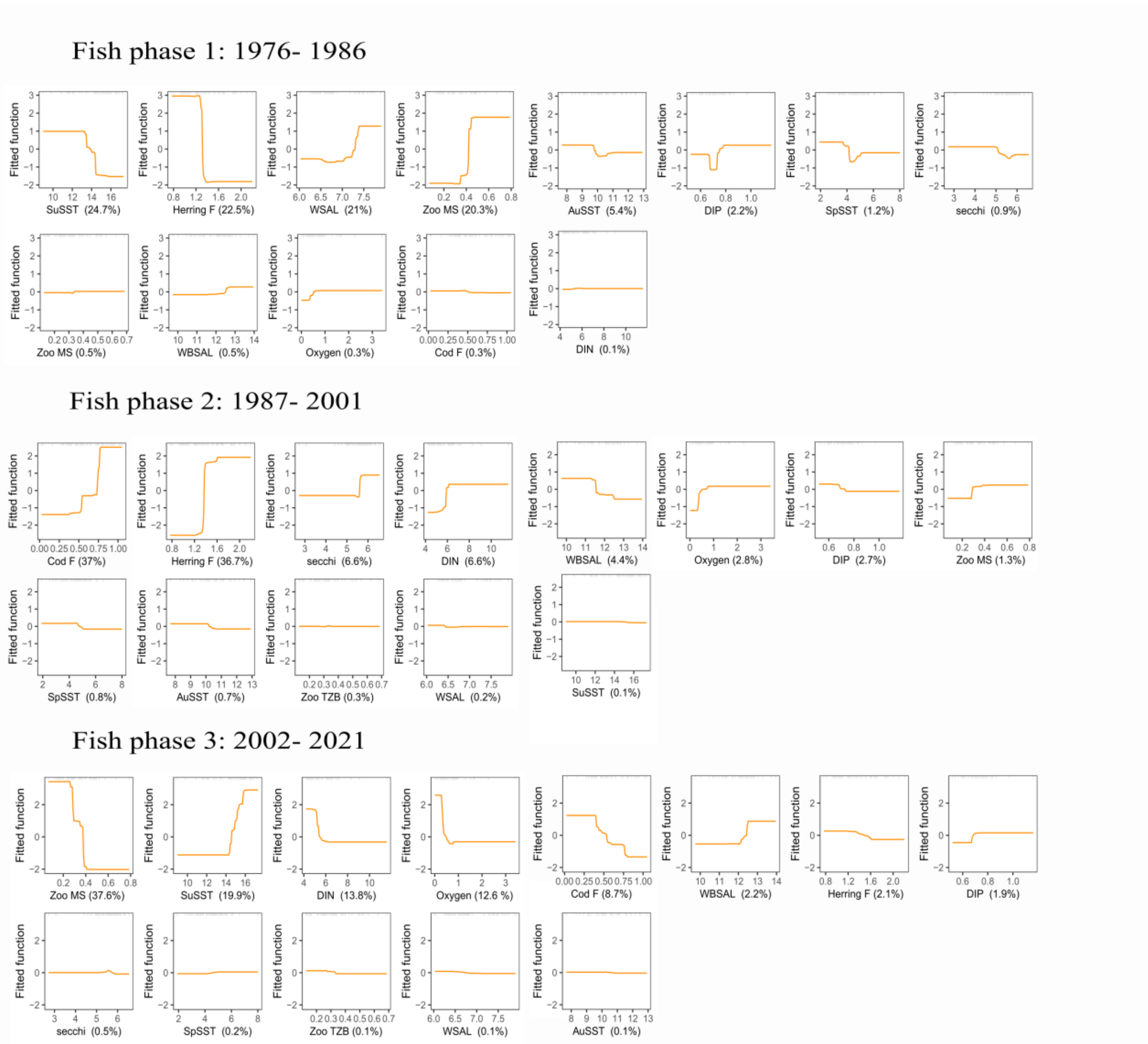

Figure.S7: Partial dependency plots for predictors of each of the clusters for fish community structure. The graphs show the effect of a given predictor on the probability of occurrence of a shift while keeping all other variables at their mean. Relative influence of each predictor is reported between parentheses.

3.3. Fish BRT results without herring fishing mortality

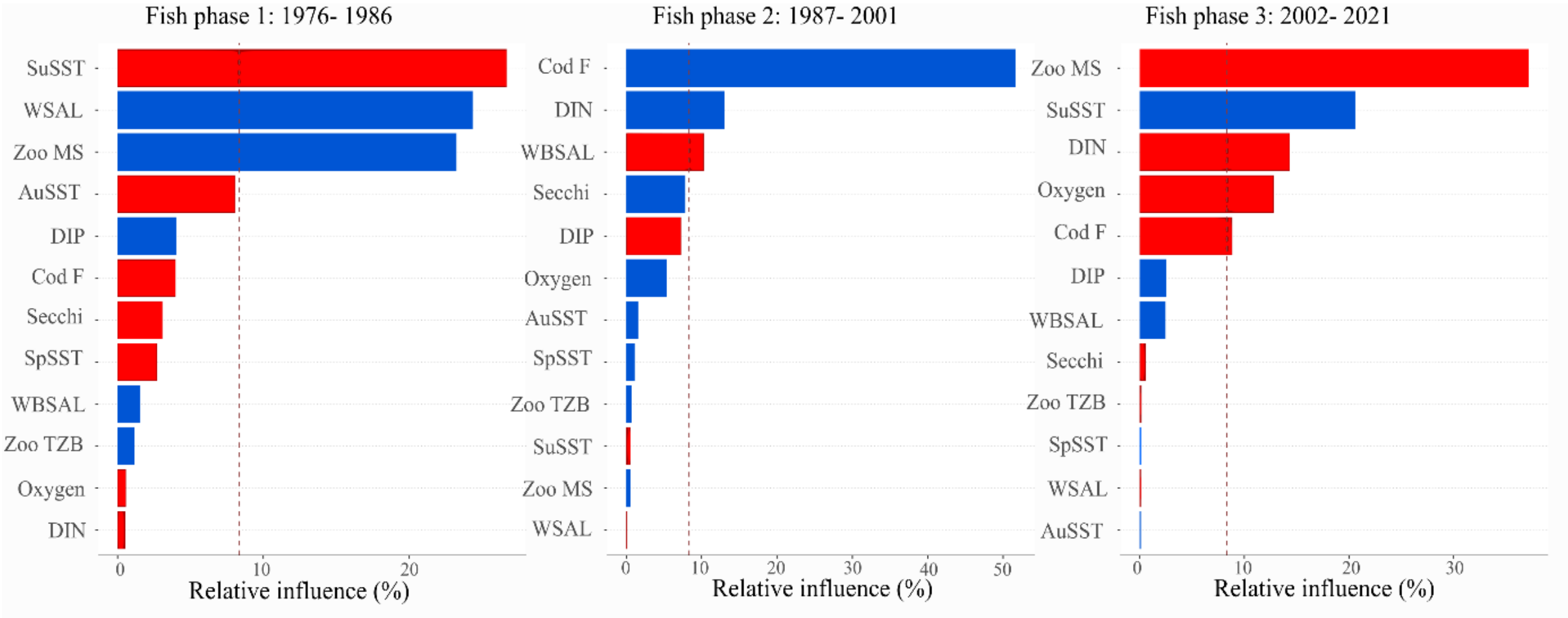

Figure.S8: Analysis results of the fish community structure without herring fishing mortality. Relative influence of drivers/pressures during each of the phases identified with BRT analysis. The colors of the bars reflect the direction of the relationship, with blue being positive, red being negative, and grey indicating a lack of clear direction. The dotted line indicates a relative influence above what could be expected by chance and is considered a driver (Jouffray et al. 2019).

When removing the herring fishing mortality, the driver was identified as similar in the first and third phases but different in the second phase (Figure S8). In the second phase, we identify cod fishing mortality and dissolved inorganic nitrogen with positive directions of the relationship, and winter bottom salinity (WBSAL) with a negative direction of the relationship. This indicates that lower bottom salinity contributes to driving the decrease in cod stock, characterizing the second phase. Please note that the performance metrics are lower than when counting herring fishing mortality as a driver, although still acceptable (Table.S3).

Table.S3: Performance metrics and cross-validation (CV) statistics for the fish community structure boosted regression tree (BRT) analysis. Tree complexity = 5, learning rate = 0.001, bag fraction = 0.75.

Performance metrics and cross validation	Phase 1	Phase 2	Phase 3
Total Deviance	1.10	1.26	1.37
Residual Deviance	0.18	0.22	0.02
Correlation	0.97	0.97	1
AUC	1	1	1
Percentage of Explained Deviance	83.43	82.34	98.72
CV Deviance	0.7	0.7	0.39
CV Correlation	0.71	0.73	0.87
CV AUC	0.94	0.95	0.97
CV Percentage of Explained Deviance	36.27	44.38	71.41

References

Ammar, Yosr, Suzanne Faxneld, Martin Sköld, and Anne L. Soerensen. 2024. "Long-Term Dataset for Contaminants in Fish, Mussels, and Bird Eggs from the Baltic Sea." *Scientific Data* 11 (1): 400. https://doi.org/10.1038/s41597-024-03216-0.

Danielsson, C., K. Wiberg, P. Korytar, S. Bergek, U. A. Th Brinkman, and P. Haglund. 2005. "Trace Analysis of Polychlorinated Dibenzo-p-Dioxins, Dibenzofurnas and WHO Polychlorinated Biphenyls in Food Using Comprehensive Two-Dimensional Gas Chromatography with Elctron-Capture Detection." *Journal of Chromatography. A, Including Electrophoresis and Other Separation Methods* 1086 (1–2): 61–70. https://doi.org/10.1016/j.chroma.2004.11.071.

Jouffray, Jean-Baptiste, Lisa M. Wedding, Albert V. Norström, Mary K. Donovan, Gareth J. Williams, Larry B. Crowder, Ashley L. Erickson, et al. 2019. "Parsing Human and Biophysical Drivers of Coral Reef Regimes." *Proceedings of the Royal Society B: Biological Sciences* 286 (1896): 20182544. https://doi.org/10.1098/rspb.2018.2544.

Liljelind, Per, Gunilla Söderström, Björn Hedman, Stina Karlsson, Lisa Lundin, and Stellan Marklund. 2003. "Method for Multiresidue Determination of Halogenated Aromatics and PAHs in Combustion-Related Samples." *Environmental Science & Technology* 37 (16): 3680–86. https://doi.org/10.1021/es0263994.

Wiberg, Karin, Michael Oehme, Peter Haglund, Heidi Karlsson, Mats Olsson, and Christoffer Rappe. 1998. "Enantioselective Analysis of Organochlorine Pesticides in Herring and Seal from the Swedish Marine Environment." *Marine Pollution Bulletin* 36 (5): 345–53. https://doi.org/10.1016/S0025-326X(97)00187-2.
